# The Energy of Muscle Contraction. III. Kinetic Energy During Cyclic Contractions

**DOI:** 10.3389/fphys.2021.628819

**Published:** 2021-04-07

**Authors:** Stephanie A. Ross, Sebastián Domínguez, Nilima Nigam, James M. Wakeling

**Affiliations:** ^1^Neuromuscular Mechanics Laboratory, Department of Biomedical Physiology and Kinesiology, Simon Fraser University, Burnaby, BC, Canada; ^2^Department of Mathematics, Simon Fraser University, Burnaby, BC, Canada; ^3^Department of Mathematics and Statistics, University of Saskatchewan, Saskatoon, SK, Canada

**Keywords:** skeletal muscle, muscle mechanics, muscle mass, finite element method, inertia, cyclic contractions

## Abstract

During muscle contraction, chemical energy is converted to mechanical energy when ATP is hydrolysed during cross-bridge cycling. This mechanical energy is then distributed and stored in the tissue as the muscle deforms or is used to perform external work. We previously showed how energy is distributed through contracting muscle during fixed-end contractions; however, it is not clear how the distribution of tissue energy is altered by the kinetic energy of muscle mass during dynamic contractions. In this study we conducted simulations of a 3D continuum muscle model that accounts for tissue mass, as well as force-velocity effects, in which the muscle underwent sinusoidal work-loop contractions coupled with bursts of excitation. We found that increasing muscle size, and therefore mass, increased the kinetic energy per unit volume of the muscle. In addition to greater relative kinetic energy per cycle, relatively more energy was also stored in the aponeurosis, and less was stored in the base material, which represented the intra and extracellular tissue components apart from the myofibrils. These energy changes in larger muscles due to greater mass were associated lower mass-specific mechanical work output per cycle, and this reduction in mass-specific work was greatest for smaller initial pennation angles. When we compared the effects of mass on the model tissue behaviour to that of *in situ* muscle with added mass during comparable work-loop trials, we found that greater mass led to lower maximum and higher minimum acceleration in the longitudinal (*x*) direction near the middle of the muscle compared to at the non-fixed end, which indicates that greater mass contributes to tissue non-uniformity in whole muscle. These comparable results for the simulated and *in situ* muscle also show that this modelling framework behaves in ways that are consistent with experimental muscle. Overall, the results of this study highlight that muscle mass is an important determinant of whole muscle behaviour.

## Introduction

Skeletal muscles are the motors that drive human and animal locomotion. Yet despite their fundamental importance, our understanding of whole muscle behaviour is relatively limited due to practical and ethical considerations that hinder accurate *in vivo* measures. To estimate the behaviour of whole muscle, measures of single fibres, fibre bundles, or small whole muscles during controlled, maximal contractions are often extrapolated to larger sizes by scaling the forces with cross-sectional area and the lengths and velocities with optimal length (Zajac, 1989). Because the effects of mass in maximally active single fibres or fibre bundles are likely negligibly small, the effects of muscle mass are not accounted for in this common method of scaling. As a consequence, muscle mass is not accounted for in estimates of larger whole muscle behaviour where the effects of mass are likely not negligible.

Muscle mass is also important for understanding whole muscle behaviour in small animals, particularly when the muscle is contracting submaximally. Josephson and Edman (1988) examined the maximum shortening speed of different fibre-types and found that fast fibres contract slower when within a fibre bundle than when in isolation. The authors suggested that the load of surrounding bundle fibres acts to slow the maximum contraction speed of the fast fibres. More recently, Holt et al. (2014) found that whole rat plantaris muscle reaches slower maximum contraction speeds when submaximally compared to maximally active, regardless of the fibre-type composition of the active tissue. The authors suggested that inactive tissue during submaximal contractions may act to slow the maximum speed of active fibres. This conclusion was supported by a simulation study that used a mass-enhanced Hill-type muscle model to replicate the contractile conditions in Holt et al. (2014) and showed that the mass of inactive fibres slows the maximum contraction speeds of whole muscle (Ross and Wakeling, 2016).

Due to challenges in experimentally manipulating muscle mass and controlling for differences in geometry and architecture across animals of different sizes, most of our understanding of the effects of muscle mass is from simulations using one-dimensional (1D) Hill-type models that account for distributed tissue mass. These studies have shown that the greater mass of larger muscles decreases the rate of muscle force development (Günther et al., 2012) and maximum contraction velocity (Ross and Wakeling, 2016) compared to smaller muscles. More recently, we showed that greater muscle mass decreases the mass-specific mechanical work and average power per cycle during cyclic contractions (Ross et al., 2018a), a finding we later supported with *in situ* experiments on rat plantaris muscle (Ross et al., 2020). However, when we compared the experimental results to simulations of the mass-enhanced 1D Hill-type model, we found that the reductions in mass-specific work were greater for the experimental compared to the simulated muscle. It may be that the three-dimensional (3D) structure of the *in situ* muscle contributed to this discrepancy between the experimental and simulated mass effects.

Although whole muscles are often modelled as 1D, 3D tissue structure has important implications for muscle mechanical behaviour. When muscles contract and shorten in length, they bulge in width or depth to maintain a nearly constant volume (Zuurbier and Huijing, 1993; Böl et al., 2013; Randhawa and Wakeling, 2015), which causes energy to be stored in the tissue as it deforms (Wakeling et al., 2020). Because muscle tissue displaces in transverse directions during contraction, muscle tissue mass can be accelerated transversely, unlike in 1D mass-enhanced Hill-type models where displacements, velocities, and accelerations can only occur in the longitudinal direction. While studies using 3D continuum muscle models have shown that muscle mass decreases the rate of force development (Böl and Reese, 2008), maximum shortening speed (Meier and Blickhan, 2000; Böl and Reese, 2008), and mass-specific mechanical work per cycle (Ross et al., 2018a), it is not clear if these changes in output muscle behaviour are related to changes in tissue energy storage and distribution as a consequence of muscle mass.

In the first two papers in this series (Ryan et al., 2020; Wakeling et al., 2020), we showed that the internal energy distribution through muscle tissue is related to the 3D behaviour of whole muscle, but to date the role of internal kinetic energy has not been considered. Muscle has internal kinetic energy during dynamic contractions, due to the presence and velocity of distributed tissue mass. In this study we explore how muscle mass and its kinetic energy influence how energy is distributed through whole pennate muscle tissue, and how this energy distribution is related to 3D tissue deformations, as well as the mechanical work done during cyclic contractions. To accomplish this, we simulated cyclic contractions of a 3D continuum muscle model with bursts of activation timed to sinusoidal length changes to mimic the experimental work-loop paradigm (Josephson, 1985). We examined the distribution of tissue energy and external mechanical work per cycle across a range of strain amplitudes, maximum excitations, and initial fibre pennation angles. To qualitatively validate the effects of muscle mass and strain amplitude on contractile behaviour, we additionally compared the tissue accelerations in the model to data collected on *in situ* rat plantaris muscle during comparable work-loop trials.

## Materials and Methods

### 3D Muscle Model

To explore the role of muscle mass on the distribution of tissue energy and external work done by muscle during contraction, we used a 3D continuum model of whole muscle that accounts for distributed tissue mass. We modelled the muscle as a fibre-reinforced composite biomaterial, in which the model fibres represented the contractile elements, or myofilaments of the muscle fibres. These fibres were embedded in a base or background material that represented the additional tissue within and surrounding the muscle fibres, including the extracellular matrix, connective tissue, fat, and blood. The muscle model fibres only generated force along their length and the base material acted in all directions, which resulted in an overall anisotropic response of the muscle tissue.

We modelled the muscle fibre stress using a similar formulation as a 1D Hill-type model, in which the fibre stress depended on the active stress-stretch, active stress-strain rate, and passive stress-stretch relationships. We modelled the muscle base material as a non-linear and isotropic elastic material such that the base material stress depended only on the passive stress-stretch properties (Yeoh, 1993). As for the magnetic resonance imaging-derived geometries in Wakeling et al. (2020), we accounted for the effects of aponeuroses on whole muscle behaviour. We modelled the aponeurosis tissue as a fibre-reinforced composite biomaterial in which the embedded fibres represented collagen fibres and only generated passive stress, unlike the activatable muscle fibres. The aponeurosis base material acted as an isotropic non-linear elastic material that was stiffer than the muscle base material. For a more detailed description of the tissue material properties used in our model, consult Wakeling et al. (2020).

Our previous 3D muscle model (Wakeling et al., 2020) was quasistatic and did not account for the effects of muscle mass, or the effects of local strain rate on muscle fibre force. In addition to considering the stress-strain rate effects to the muscle fibre response (Ross et al., 2018b) in our current model, we also considered the kinetic and internal energies and accounted for the external work done on the system. Note that both the kinetic and internal energies depended on the velocity, unlike our previous model in Wakeling et al. (2020) and Ryan et al. (2020) which assumed quasistatic deformations. The equations for the momentum and mass balance are described in the Appendix and detailed in Ogden (1984). The Hill-type stress-stretch and stress-strain rate relations cannot be obtained through standard variations of the energy; instead, these relations were used to define the constitutive laws. We used the energy as a post-processed quantity, consistent with our approach in Wakeling et al. (2020) and Ryan et al. (2020).

We approximated the solutions of the momentum and mass balance equations (see Appendix) using a semi-implicit time-stepping method in which we post-processed the velocity based on the implicit computation of the displacement, pressure, and dilation via the finite element method, similar to our previous approach (Wakeling et al., 2020).

### Muscle Model Geometries

**FIGURE 1:**
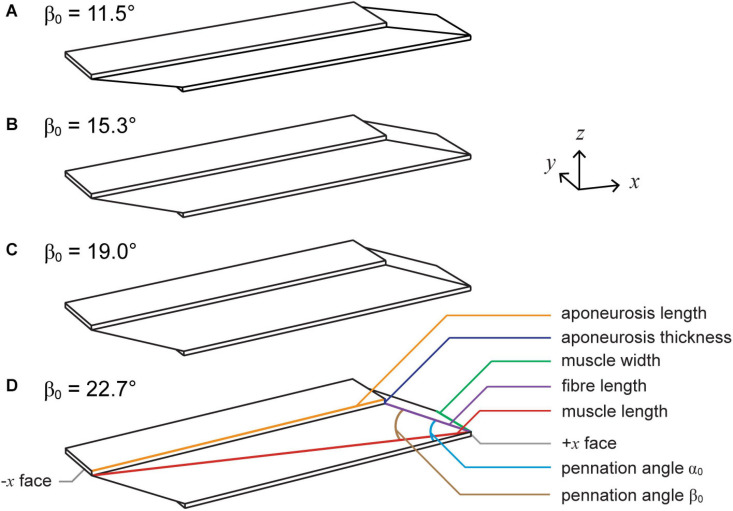
Muscle geometries. We simulated muscle with initial pennation angles relative to the bottom aponeurosis α_0_ of 15° **(A)**, 20° **(B)**, 25° **(C)**, and 30° **(D)**, which resulted in initial pennation angles relative to the –*x* axis β_0_ of 11.5°, 15.3°, 19.0°, and 22.7°. To vary α_0_, we altered the initial fibre length and kept the aponeurosis length, aponeurosis thickness, muscle width, and muscle length constant.

We constructed the root muscle geometry to represent the approximate size and proportions of a human medial gastrocnemius muscle (Randhawa et al., 2013). The muscle fibres were oriented in the *xz* plane with an initial pennation angle α_0_ of 20° relative to the bottom (−*z*) aponeurosis (Figure 1). This resulted in a pennation angle β_0_ of 15.3° relative to the −*x* axis or the longitudinal direction. In this paper we varied the angle of the geometry with α_0_ of the fibres, unlike in Wakeling et al. (2020) and Ryan et al. (2020) in which we varied β_0_ of the fibres within blocks of muscle tissue. To vary α_0_ from the root geometry, we varied the initial fibre length and kept the initial aponeurosis length, aponeurosis thickness, muscle length in the *x* direction, and muscle width in the *y* direction constant. We examined geometries with α_0_s of 15, 20, 25, and 30°, which resulted in β_0_s of 11.5, 15.3, 19.0, and 22.7°. See Table 1 for definitions of all variables used in the main text and Appendix Table A1 for details of the initial dimensions of the muscle geometries.

**TABLE 1:** Symbols and definitions of variables in the main text.

**Symbol**	**Definition**
**u**	Displacement vector
ψ	Strain energy-density
$\bar{\psi }$	Mean strain energy-density
${\bar{\psi }}_{\text{apo}}$	Mean aponeurosis strain energy-density
${\bar{\psi }}_{\text{muscle},\text{base}}$	Mean muscle base material strain energy-density
${\bar{\psi }}_{\text{muscle},\text{act}}$	Mean muscle active strain energy-density
${\bar{\psi }}_{\text{muscle},\text{pas}}$	Mean muscle passive strain energy-density
${\bar{\psi }}_{\text{muscle},\text{vol}}$	Mean muscle volumetric strain energy-density
${\bar{\psi }}_{\text{kin}}$	Mean kinetic energy-density
*ê*_max_	Normalized maximum excitation
α_0_	Pennation angle relative to bottom aponeurosis in the initial configuration
β_0_	Pennation angle relative to *x*-axis in the initial configuration
$\bar{\beta }$	Mean pennation angle relative to *x*-axis in the current configuration
${\bar{\lambda }}_{\text{tot}}$	Total mean fibre stretch
*l*	Muscle length
ε_max_	Maximum strain amplitude of sinusoidal muscle length changes
*F*_*x*_	Force in *x*-direction on +*x* face of bottom (−*z*) aponeurosis
W*	Mass-specific mechanical work per cycle
a_mid_	Relative acceleration in the *x*-direction over time of a quadrature point near the middle of the model or of the pin inserted near the middle of the *in situ* muscle
a_end_	Relative acceleration in the *x*-direction over time at the end of the model or the *in situ* muscle
a_max,end_	Maximum a_end_ for a given simulation or experimental trial
a_max,mid_	Maximum a_mid_ for a given simulation or experimental trial
a_min,end_	Minimum a_end_ for a given simulation or experimental trial
a_min,mid_	Minimum a_mid_ for a given simulation or experimental trial

To explore the effects of muscle mass across a range of muscle sizes, we geometrically scaled the human medial gastrocnemius-sized geometries to 2.5 and 3.5 times the size (length scale factor or “scale”) to approximate the behaviour of muscles in larger animals (Alexander et al., 1981). This resulted in initial muscle lengths, widths, and thicknesses that varied with the scale, areas that varied with the scale-squared, and volumes that varied with the scale-cubed. Because the initial density was constant at 1060 kg m^–3^ (Méndez and Keys, 1960), muscle mass (including that of the aponeurosis) also varied with the scale-cubed, as with the volume. This method of scaling is described in more detail in Ross et al. (2018b), although the scale 1 geometry in this study is the approximate size of a human medial gastrocnemius, whereas in our previous studies it was the size of a fibre bundle (Ross et al., 2018a,b).

### Simulations and Post-processing

**FIGURE 2:**
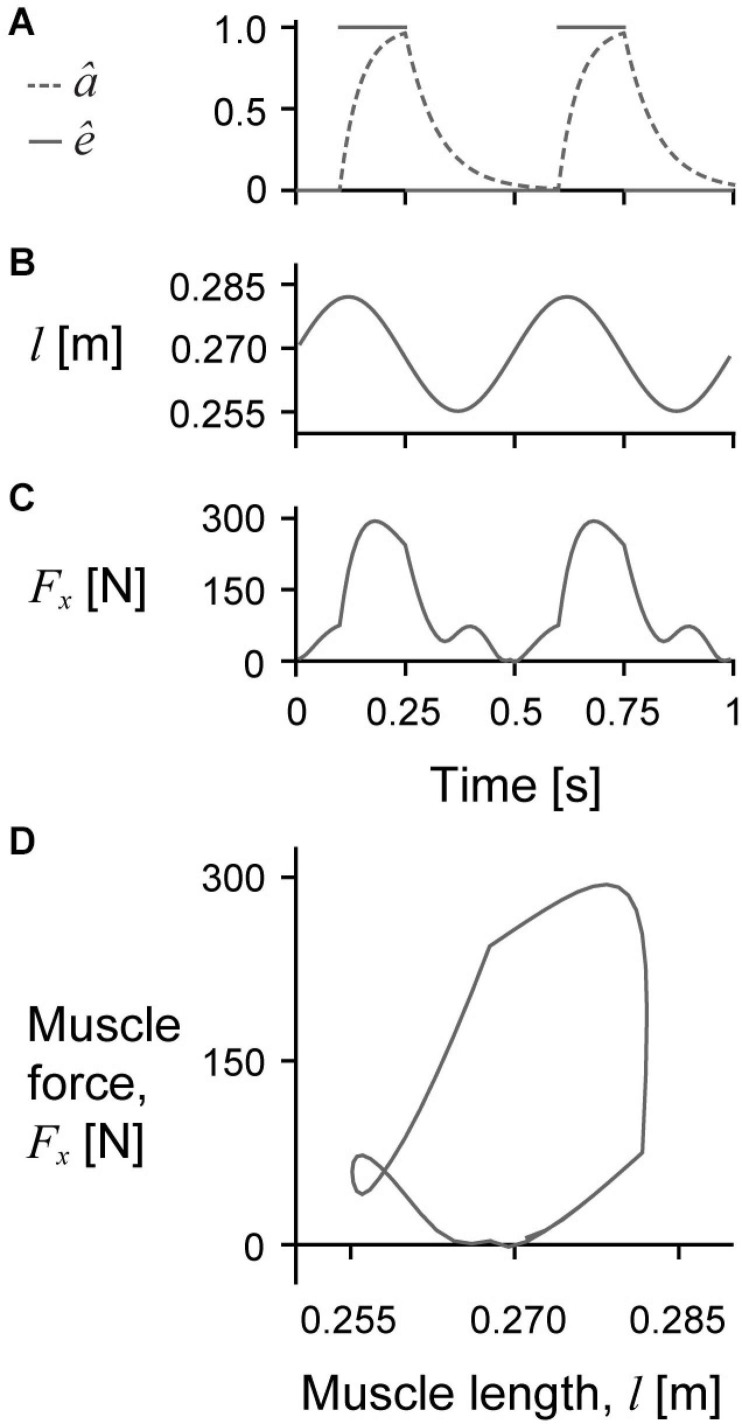
Sample raw simulation traces. Raw traces of normalised excitation *ê* (solid lines) and activation *â* (dashed line) over time **(A)**, muscle length *l* over time **(B)**, muscle force *F*_*x*_ over time **(C)**, and *F*_*x*_ over *l* in which the area inside the loop represents the mechanical work per cycle **(D)** for a representative simulation with a cycle strain amplitude of 5%, maximum normalised excitation of 1, initial pennation β_0_ of 15.3°, and scale of 1.

We simulated cyclic work-loop regimes of the muscle model in which the total muscle length followed a sinusoidal trajectory timed with bursts of excitation (Josephson, 1985). To achieve the sinusoidal length changes, we fixed the −*x* face of the top (+*z*) aponeurosis in all directions and applied a non-zero Dirichlet boundary condition to the +*x* face of the bottom (−*z*) aponeurosis to constrain it to follow a sinusoidal trajectory in the *x* direction and remain fixed in the *z* and *y* directions. All other surfaces of the model remained unconstrained throughout the simulations. To mimic the nerve stimulation in *in situ* work-loop experiments, we cyclically excited the muscle using a square wave excitation trace where the excitation was either 0 or the maximum excitation *ê*_max_ (Figure 2). Note that we chose to use *ê*_max_ to represent the maximum excitation instead of *u*_max_ from our previous work (Ross et al., 2018a,b) to avoid confusion with the displacement **u**. We then converted the excitation to activation using the excitation-activation transfer function from Zajac (1989). As with typical work-loop experiments, we excited the muscle with only one burst of excitation per length cycle such that both the sinusoidal length changes and excitations had a frequency of 2 Hz. To match the experimental data collected on *in situ* muscle (described later), each excitation burst started 5% in time before the start of shortening and continued for 30% of the length cycle duration (duty cycle of 0.3). These contractile conditions resulted in the muscle generating active force primarily while shortening. For β_0_ of 11.5, 19.0, and 22.7°, we set *ê*_max_ to 1 and the maximum strain amplitude ε_max_ to 5% of resting optimal length for a total shortening strain of 10%. For β_0_ of 15.3°, we also examined submaximal activation with *ê*_max_ of 0.4 in addition to maximal activation with *ê*_max_ of 1. We also examined ε_max_ of 2.5% and 7.5% in addition to 5% for β_0_ of 15.3° to explore how ε_max_ alters the effects of muscle mass during cyclic contractions.

To quantify the energy distribution through the muscle tissue, we used the strain energy-density ψ, which is the strain energy per unit volume of tissue in J m^–3^. ψ is given at each quadrature point in the model, so to determine the total mean ψ for the whole muscle ψ, we used the sum of the ψs at each point weighted by the volume fraction of the given point relative to the whole muscle (aponeurosis and muscle) volume. The total strain energy-density of the muscle was comprised of the aponeurosis ${\bar{\psi }}_{\text{apo}}$, muscle base material ${\bar{\psi }}_{\text{muscle},\text{base}}$, active muscle fibre ${\bar{\psi }}_{\text{muscle},\text{act}}$, passive muscle fibre ${\bar{\psi }}_{\text{muscle},\text{pas}}$, volumetric ${\bar{\psi }}_{\text{muscle},\text{vol}}$, and kinetic ${\bar{\psi }}_{\text{kin}}$ strain energy-densities. We defined ${\bar{\psi }}_{\text{apo}}$ as the sum of the aponeurosis fibre (passive only), volumetric, and base material strain energy-densities and ${\bar{\psi }}_{\text{kin}}$ as the sum of the aponeurosis and muscle kinetic strain energy-densities.

We calculated the muscle mechanical work per cycle in J as the integral of the *x*-component of the force perpendicular to the +*x* face of the bottom aponeurosis *F*_*x*_ over the muscle length *l* for one cycle. We defined *l* as the distance in the *x*-direction between the −*x* face of the top aponeurosis and the +*x* face of the bottom aponeurosis (Figure 1). Because larger muscles with larger cross-sectional areas and longer lengths will have greater work per cycle, we normalised the mechanical work per cycle in J by the mass of the given geometry to give the mass-specific mechanical work per cycle *W*^∗^ in J kg^–1^. We calculated the mass of each geometry as the product of the initial total volume in m^3^ (muscle and aponeurosis) and the initial density of 1060 kg m^–3^.

To determine how the muscle fibre stretches and pennation angles changed with scale and β_0_, we examined the total mean fibre stretch ${\bar{\lambda }}_{\text{tot}}$ and mean current fibre angle relative to the −*x* axis $\bar{\beta }$ throughout each simulation. We calculated ${\bar{\lambda }}_{\text{tot}}$ as the sum of the fibre stretches at each quadrature point weighted by the volume fraction of the given point relative to the total muscle volume, and $\bar{\beta }$ as the mean fibre angle relative to the −*x* axis.

To explore changes in the uniformity of tissue behaviour with greater muscle scale and ε_max_, we examined tissue accelerations in the *x* direction near the middle of the muscle compared to at the non-fixed end. To determine the tissue accelerations near the middle of the muscle, we identified the quadrature point closest to the middle of the geometry in the *x*, *y*, and *z* directions in the initial configuration and then tracked the position of that point in the *x*-direction throughout each simulation. We then fitted a Fourier series function with three harmonics to the position-time data, which we normalised to the distance from the fixed end to the centre quadrature point at rest (approximately 0.5 × *l*), and took the second time derivative of the fitted function to estimate the acceleration a_mid_ in s^–2^. We repeated this same process to determine the tissue accelerations at the moving end of the muscle a_end_ (+*x* face of −*z* aponeurosis), except we normalised the position data to the muscle length at rest. For a massless spring undergoing cyclic length changes, the acceleration in the *x*-direction in m s^–2^ would be smallest near the fixed end of the spring and largest near the moving end. If the acceleration was then normalised to the distance at rest between the fixed end and the location where the acceleration was measured, the acceleration in the *x*-direction in s^–2^ would be the same near the fixed end and middle as at the end of the spring. Thus, for cyclic muscle contractions a difference between a_mid_ and a_end_ indicates that the muscle tissue behaviour is not uniform along its length. Because we compared the simulation acceleration results to that of the *in situ* experiments, we only quantified a_mid_ and a_end_ for simulations with β_0_ of 15.3°, as rat plantaris muscle has a pennation angle of approximately 15–16° at rest (Roy et al., 1982; Eng et al., 2008). We also only examined simulations with *ê*_max_ of 1, as the *in situ* muscles were maximally excited during the work-loop trials.

### Experimental Data Collection

We compared the model tissue accelerations during the simulations to previously unpublished data collected on *in situ* muscle as part of a larger study (Ross et al., 2020). All experiments were conducted in accordance with the guidelines of the Faculty of Arts and Sciences Institutional Animal Care and Use Committee of Harvard University and the University Animal Care Committee of Simon Fraser University. We examined the effect of adding mass to the right plantaris muscle of seven Sprague Dawley rats [*Rattus norvegicus*; body mass: 416.3 ± 31.9 g (mean ± SD); Charles River, Wilmington, MA, United States]. For details of the experimental preparation and surgery, consult Ross et al. (2020). In brief, the animals were kept deeply anaesthetised for the duration of the experiments. We isolated and separated the plantaris muscle from underlying tissue and then cut the distal plantaris tendon and tied the distal end of the muscle to a movement arm on a servomotor (series 305B-LR; Aurora Scientific Inc., Aurora, ON, Canada). The proximal end of the muscle remained attached to the femur through the proximal tendon, and we fixed the femur using a stereotaxic frame and femur clamp. To externally stimulate the muscle, we placed a bipolar cuff electrode around the sciatic nerve and then severed the nerve proximal to the cuff to remove descending nervous control.

To alter the mass properties of the *in situ* muscle, we inserted a pin into the muscle midway along its length. This pin was attached to a movement arm, and at the other end of the arm, we attached different size weights to add effective mass to the muscle acting through the pin. We used a photodiode LED pair to track the position of the movement arm and therefore the position of the pin inserted into the muscle. We added two different effective masses to the plantaris muscles: movement arm with no weight and movement arm + 1.1 g weight, resulting in mean ± SD effective masses of 84.9 ± 7.5%, and 122.9 ± 10.8% muscle mass, respectively. As for the model simulations, we conducted work-loop trials in which we constrained the muscle length to follow a 2 Hz sinusoidal trajectory, and we supramaximally stimulated the muscle via the sciatic nerve to fully activate the muscle tissue. In addition to altering the effective mass added to the muscle, we also varied the ε_max_ of the sinusoidal muscle length trajectories (5, 7.5, and 10% of optimal length).

### Experimental Data Analysis

We filtered the output photodiode data using a 4th order Butterworth low-pass filter with a 55 Hz cut-off frequency and converted the output voltages to positions using a calibration curve. As with the simulation data, we fit Fourier series with three harmonics to discrete samples of the pin position and the end of the muscle for each trial and took the second derivative of this function with respect to time to estimate the accelerations at the pin near the centre and at the end of the muscle. The pin and end accelerations were normalised using the distance between the proximal end of the muscle that was attached to the femur and the pin at rest and the muscle length at rest, respectively. The mean ± SD distance between the distal end of the muscle and the pin at rest was 16.4 ± 2.7 mm, and between the proximal and distal ends of the muscle (muscle length) at rest was 40.3 ± 2.1 mm. We denoted the pin acceleration as a_mid_ and the acceleration at the moving end as a_end_ to provide a comparison with the simulation results.

To examine the effects of added mass and ε_max_ on internal tissue accelerations during these *in situ* work-loop trials, we conducted repeated measures analysis using a linear mixed model that we fit using maximum likelihoods with the function *lmer* in the package *lme4* (Bates et al., 2014) in R (version 3.6.1; R Core Team, 2019). We included the difference between the maximum acceleration at the end a_max,end_ and maximum acceleration near the middle a_max,mid_ of the muscle as continuous response variables, ε_max_ and added mass conditions as categorical fixed effects, and subject number as a categorical random effect in the model. To determine the pairwise differences in a_max,end_ and a_max,mid_ across all ε_max_ and added mass conditions, we used the Holm-Bonferroni method (Holm, 1979) within the package *multcomp* (Hothorn et al., 2008) to control for the increase in family-wise error rate with multiple comparisons. We repeated this analysis a second time to examine the effects of added mass and ε_max_ on the difference between the minimum acceleration at the end a_min,end_ and near the middle a_min,mid_ of the muscle.

## Results

We excited the muscle model slightly before it reached its longest length and until the middle of the shortening phase (Figure 2A), which caused the muscle to reach peak activation near the middle of the cycle. The activation decayed through the second half of the shortening phase and reached zero near the end of the cycle. Because the muscle was primarily active during shortening, the net mass-specific work per cycle *W*^∗^ was positive for all simulations (Figure 2D), although there was a short period of negative work when the muscle was producing active force during lengthening for some conditions (Figure 2C).

**FIGURE 3:**
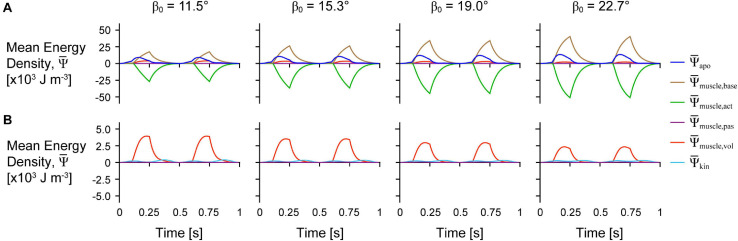
Tissue energy over time across initial pennation angles. Simulation traces of mean energy-density $\bar{\psi }$ over time for geometries with different initial pennation angles relative to the *x*-axis β_0_ at scale 1 and with a maximum strain of 5% and normalised excitation of 1. **(A)** Shows all of the energy components and **(B)** shows only the passive, kinetic, and volumetric components from **(A)** with the *y*-axis rescaled to visualise changes in these smaller components.

**FIGURE 4:**
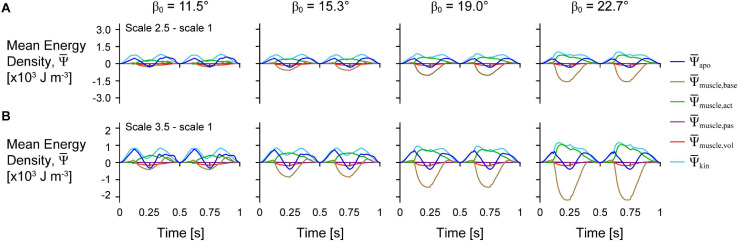
Difference in tissue energy over time between scales and across initial pennation angles. Difference in mean energy-density $\bar{\psi }$ across tissue components between scale 2.5 and scale 1 **(A)** and scale 3.5 and scale 1 **(B)** simulations. Greater scale muscles are larger in size and have greater mass. All traces are for simulations with a strain of 5%, normalised excitation of 1, and initial pennation angles β_0_ ranging from 11.5 to 22.7°.

The distribution of the strain energy-density ψ, across the different components varied over time during each cycle (Figure 3). The energy-density due to the active fibres ${\bar{\psi }}_{\text{muscle},\text{act}}$ decreased (became more negative) when the activation was greater than 0, and the other energy components increased in opposition to these decreases in ${\bar{\psi }}_{\text{muscle},\text{act}}$, particularly ${\bar{\psi }}_{\text{muscle},\text{base}}$, and ${\bar{\psi }}_{\text{apo}}$. ${\bar{\psi }}_{\text{apo}}$ increased with longer muscle lengths as the aponeurosis was stretched. Greater initial pennation angle β_0_ resulted in slightly greater increases in ${\bar{\psi }}_{\text{apo}}$, greater increases in ${\bar{\psi }}_{\text{muscle},\text{base}}$, and greater decreases in ${\bar{\psi }}_{\text{muscle},\text{act}}$, but resulted in smaller increases in ${\bar{\psi }}_{\text{muscle},\text{vol}}$ and ${\bar{\psi }}_{\text{kin}}$. Comparing the distribution of strain energy-density across scales ${\bar{\psi }}_{\text{muscle},\text{base}}$ was lower and ${\bar{\psi }}_{\text{muscle},\text{act}}$ was higher during the shortening period with greater scale and therefore greater muscle size and mass (Figure 4). Greater scale resulted in higher ${\bar{\psi }}_{\text{kin}}$ throughout the cycle and higher ${\bar{\psi }}_{\text{apo}}$ when the muscle was at its longest and shortest lengths.

**FIGURE 5:**
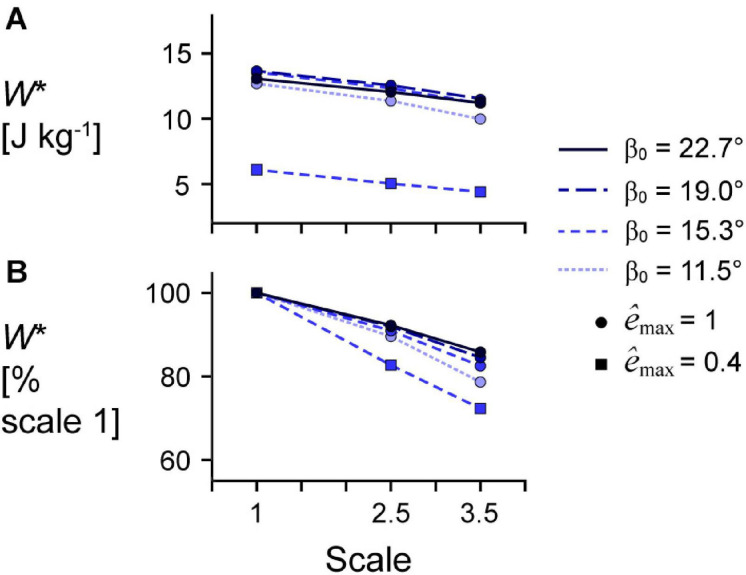
Effect of initial pennation angle on the relationship between scale and mass-specific mechanical work. **(A)** Absolute mass-specific mechanical work per cycle W* across geometry length scales (muscle sizes) for different initial pennation angles β_0_ ranging from 11.5 to 22.7° relative to the *x*-axis for maximum strain of 5% and maximum relative excitation *ê*_max_ of 1 (circles) and 0.4 (squares). W* across scales as a percentage of scale 1 W* at the same β_0_ and *ê*_max_ are shown in **(B)**. All cycles had a frequency of 2 Hz. Note that the lines connecting discrete points are intended to aid in visualization rather than to imply a linear relationship.

**FIGURE 6:**
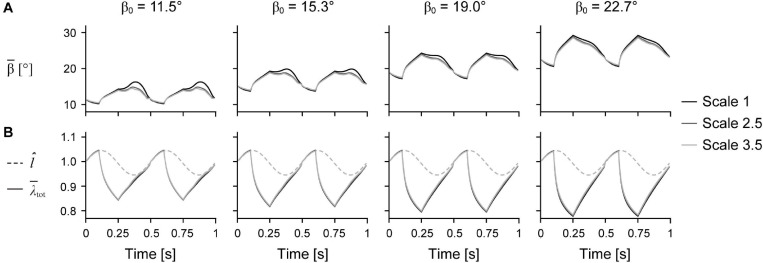
Mean fibre angle and stretch over time. Mean fibre pennation angle relative to the –*x* axis $\bar{\beta }$ **(A)** and normalised muscle length *l̂* and total mean fibre stretch ${\bar{\lambda }}_{\text{tot}}$ **(B)** over time across initial pennation angles relative to the *x*-axis β_0_ for different scales.

**FIGURE 7:**
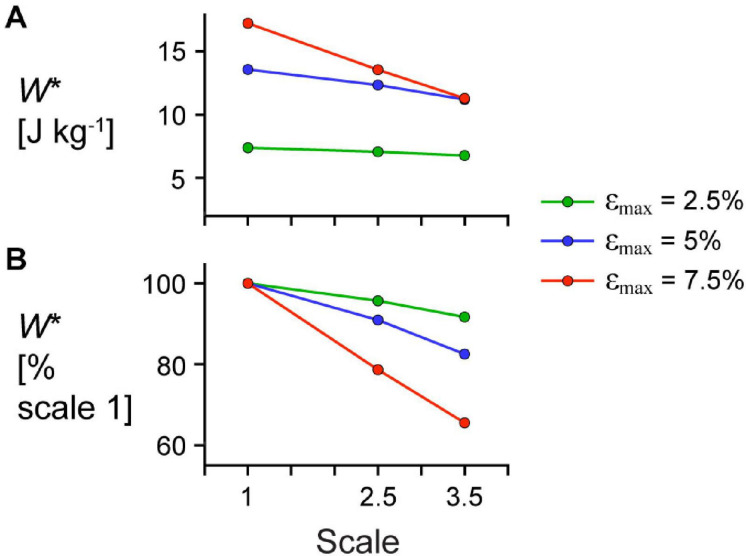
Effect of cycle strain amplitude on the relationship between scale and mass-specific mechanical work. **(A)** Absolute mass-specific mechanical work per cycle W* across scales (muscle sizes) for different maximum strain amplitudes ε_max_ and **(B)** W* as a percentage W* for scale 1 simulations at the same ε_max_. All W* results are for simulations with a maximum relative excitation of 1, initial pennation angle β_0_ of 15.3°, and 2 Hz cycle frequency. Note that the lines connecting discrete points are intended to aid in visualization rather than to imply a linear relationship.

Larger scale, and therefore greater muscle mass, resulted in lower *W*^∗^ (Figure 5). Specifically, we found a mean reduction in *W*^∗^ of 8.8% for scale 2.5 relative to scale 1, and 17.1% for scale 3.5 relative to scale 1, across β_0_s for simulations with *ê*_max_ of 1 and ε_max_ of 5%. Lower *ê*_max_ for the β_0_ of 15.3° simulations resulted in lower absolute *W*^∗^ and relative *W*^∗^ with greater scale compared to higher *ê*_max_, indicating that muscle mass had a greater effect when *ê*_max_ was lower. Higher β_0_ resulted in greater *W*^∗^ at the same scale (Figure 5A) and smaller reductions in *W*^∗^ with greater scale (Figure 5B). For simulations with *ê*_max_ of 1, β_0_ of 22.7° resulted in a 2.7% and 7.2% smaller reduction in *W*^∗^ compared to β_0_ of 11.5° at scale 2.5 and scale 3.5 relative to scale 1, respectively. Greater β_0_ also led to greater mean fibre stretches ${\bar{\lambda }}_{\text{tot}}$ and greater changes in $\bar{\beta }$ (Figure 6). Larger scale resulted in smaller changes in $\bar{\beta }$ over the cycle but nearly the same ${\bar{\lambda }}_{\text{tot}}$. Higher β_0_ led to smaller differences in $\bar{\beta }$ with greater scale. Greater cycle strain amplitude ε_max_ resulted in greater absolute *W*^∗^ for simulations with β_0_ of 15.3° and *ê*_max_ of 1 at scale 1 (Figure 7A). At scale 2.5 and 3.5, ε_max_ of 2.5% resulted in the lowest *W*^∗^ and ε_max_ of 5% resulted in the highest *W*^∗^. In terms of the effect of ε_max_ across scales, greater ε_max_ led to lower relative *W*^∗^ with greater scale (Figure 7B).

**FIGURE 8:**
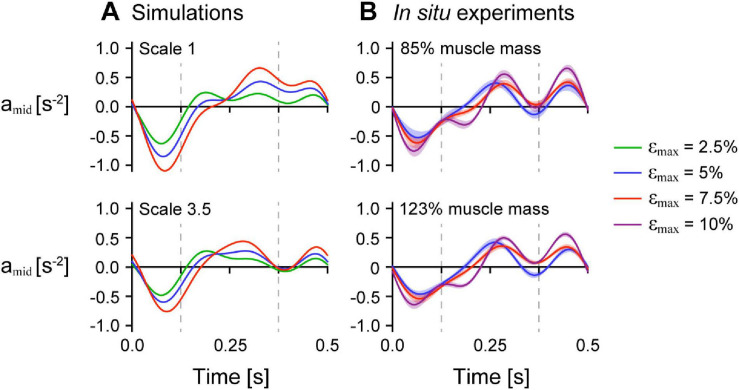
Normalised acceleration midway along the muscle’s length over time across strain conditions. Normalised tissue acceleration in the *x*-direction of a point midway along the muscle’s length a_mid_ over time for simulations with initial pennation angle β_0_ of 15.3° **(A)** and *in situ* experiments **(B)**. The dashed vertical bars at 0.125 s and 0.375 s indicates the times when the muscle was at its longest and shortest lengths, respectively. a_mid_ was normalised using the *x* distance between the centre point and the moving end of the muscle at rest for the simulations and between the inserted pin and the moving end of the *in situ* muscle at rest for the experiments. The maximum normalised excitation was 1 for the simulations and the *in situ* muscle was supramaximally stimulated to activate all the muscle fibres. The shaded intervals in **(B)** show the standard error of the mean at each time point.

To examine the effects of mass on the uniformity of tissue behaviour across the muscle, we compared the tissue accelerations at a single point near the middle of the muscle a_mid_ compared to at the moving (+*x*) end a_end_ for simulations with *ê*_max_ of 1 and β_0_ of 15.3° to compare with the experiments on maximally active *in situ* muscle. We normalised a_mid_ to the *x* distance between the fixed end and the middle point at rest and a_end_ to the muscle length at rest to give accelerations in s^–2^. Note that because the position of the end of both the simulated and *in situ* muscle was constrained to follow a sinusoidal trajectory, a_end_ did not vary across simulations and trials with the same ε_max_. For both the simulations and *in situ* experiments, a_mid_ decreased initially when the whole muscle length increased (Figure 8). The minimum acceleration of the point near the middle of the muscle a_min,mid_ occurred earlier than the minimum acceleration at the end a_min,end_ across all strain and scale and added mass conditions and was lower with greater ε_max_. The time when a_mid_ was zero and the muscle shortening velocity would be maximal occurred earlier than for a_end_ and was earlier with lower ε_max_, greater scale for the simulations, and greater added mass for the *in situ* experiments.

**FIGURE 9:**
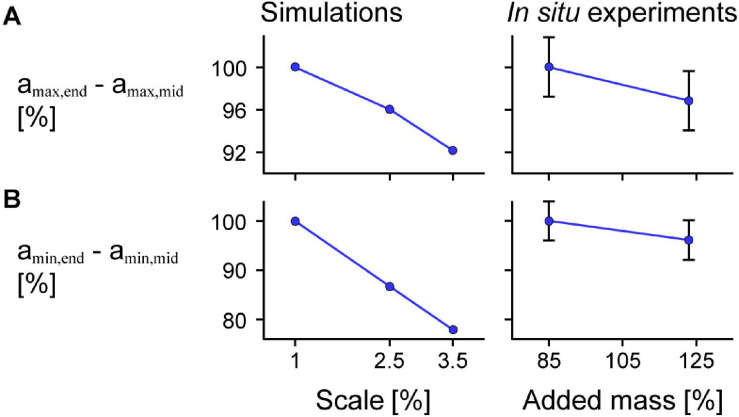
Difference in maximum and minimum acceleration near the middle compared to at the end of the muscle across scales and added masses. Mean difference in maximum normalised acceleration in the *x*-direction at the end a_max,end_ compared to that of a point near the middle of the muscle a_max,mid_ across scales (muscle sizes) for simulations relative to scale 1 (left) and *in situ* experiments relative to 85% added muscle mass relative to the muscle mass (right; *p* = 0.0056) calculated across all maximum strains **(A)**. **(B)** Shows the mean difference in minimum acceleration a_min,end_ in the *x*-direction at the end compared to near the middle of the muscle a_min,mid_ (*p* = 0.0076 for the *in situ* experiments). a_max,end_ and a_min,end_ were normalised to the muscle optimal length and a_max,mid_ and a_min,mid_ were normalised to the *x* distance between the centre point and the moving end of the muscle at rest for the simulations and between the inserted pin and the moving end of the *in situ* muscle at rest for the experiments. Although we show similar trends for greater added mass as for greater scale, we caution against directly comparing the magnitude of effects as altering the added mass and scale manipulate the tissue mass in different ways. The initial pennation angle β_0_ was 15.3° and the maximum normalised excitation was 1 for the simulations and the *in situ* muscle was supramaximally stimulated to activate all the muscle fibres. The error bars for the *in situ* experiments represent the standard error of the mean. Note that the lines connecting discrete points are intended to aid in visualization rather than to imply a linear relationship.

For the model simulations, the reduction in a_max,mid_ relative to a_max,end_ was greater for larger scales (Figure 9A). We also found similar results for the *in situ* contraction cycles where greater added mass resulted in greater reductions in a_max,mid_ relative to a_max,end_ (*p* = 0.0056). Specifically, the reduction in a_max,mid_ compared to a_max,end_ was 0.063 s^–2^ (3.1%) greater for the 123% added mass condition compared to the 85% added mass condition (SE = 0.023 s^–2^). Similar to the difference in a_max_, we also found greater increases (closer to zero) in a_min,mid_ relative to a_min,end_ for simulations in which the muscle was larger (Figure 9B) and for *in situ* experiments in which greater effective mass was added to the muscle (*p* = 0.0076). Specifically, the increase in a_min,mid_ compared to a_min,end_ was 0.073 s^–2^ (3.9%) greater for the 123% compared to the 85% added mass condition (SE = 0.028 s^–2^). While the effect of greater mass was similar for the simulations as for the *in situ* experiments, because we used different paradigms to manipulate the mass in that we geometrically scaled the simulated muscle to larger sizes and added effective mass acting through a pin inserted into the *in situ* muscle, the simulation and *in situ* mass effect results are not directly comparable.

**FIGURE 10:**
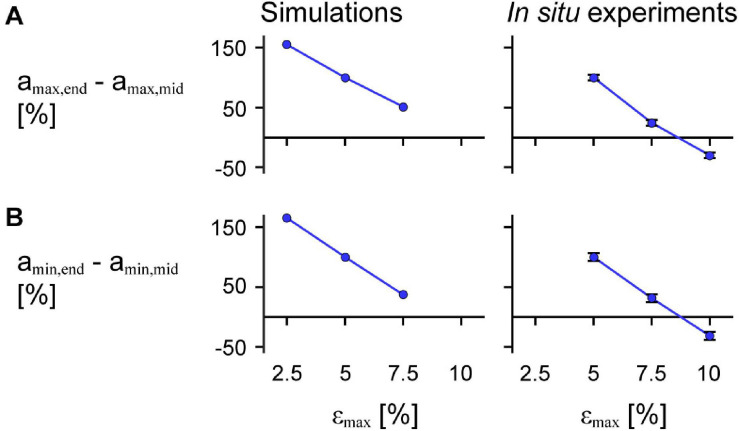
Difference in maximum and minimum acceleration near the middle compared to at the end of the muscle across maximum strains. Mean difference in maximum normalised acceleration in the *x*-direction at the end a_max,end_ compared to that of a point near the middle of the muscle a_max,mid_ across cycle strains for simulations relative to scale 1 (left) and *in situ* experiments relative to 85% added muscle mass (right; *p* < 0.001) calculated across all scales and added masses **(A)**. **(B)** Shows the mean difference in minimum acceleration a_min,end_ in the *x*-direction at the end compared to near the middle of the muscle a_min,mid_ (*p* < 0.001 for the *in situ* experiments). a_max,end_ and a_min,end_ were normalised to the muscle optimal length. a_max,mid_ and a_min,mid_ were normalised to the *x* distance between the centre point and the moving end of the muscle at rest for the simulations and between the inserted pin and the moving end of the *in situ* muscle at rest for the experiments. The initial pennation angle β_0_ was 15.3° and the maximum normalised excitation was 1 for the simulations and the *in situ* muscle was supramaximally stimulated to activate all the muscle fibres. The error bars for the *in situ* experiments represent the standard error of the mean. Note that the lines connecting discrete points are intended to aid in visualization rather than to imply a linear relationship.

We found that greater ε_max_ of the imposed motion at the end of the muscle led to greater decreases in a_max,mid_ relative to a_max,end_ for both the model simulations and *in situ* experiments (Figure 10A; *p* < 0.001). For the *in situ* muscle trials, there was a 0.91 s^–2^ (75.5%) greater reduction in a_max,mid_ relative to a_max,end_ for ε_max_ of 7.5% compared to 5% (SE = 0.027 s^–2^, *p* < 0.001), and a 0.65 s^–2^ (30.9%) greater reduction for ε_max_ of 10% compared to 7.5% (SE = 0.028 s^–2^, *p* < 0.001). Finally, we found a 1.56 s^–2^ (129.7%) greater reduction a_max,mid_ relative to a_max,end_ for ε_max_ of 10% compared to 5% (SE = 0.028 s^–2^, *p* < 0.001). We found that greater ε_max_ led to greater increases (closer to zero) in a_max,mid_ relative to a_max,end_ for both the simulations and *in situ* experiments (Figure 10; *p* < 0.001). For the *in situ* experiments, we found a 0.79 s^–2^ (68.5%) greater increase in the difference in a_min_ for 7.5% ε_max_ compared to 5% (SE = 0.033 s^–2^, *p* < 0.001), a 0.73 s^–2^ (37.3%) greater increase for 10% ε_max_ compared to 7.5% (SE = 0.034 s^–2^, *p* < 0.001), and a 1.51 s^–2^ (131.1%) greater increase for 10% ε_max_ compared to 5% (SE = 0.034 s^–2^, *p* < 0.001). While we examined different ranges of ε_max_ for the experimental trials and simulations, the differences in a_max,mid_ compared to a_max,end_ relative to 5% ε_max_ were of similar magnitude for the *in situ* experiments and simulations.

## Discussion

### Tissue Energy Distribution in Whole Muscle During Cyclic Contractions

Muscles in living animals are activated by motor neurons that carry impulses from the central nervous system. These impulses cause myosin and actin filaments to bind and form cross-bridges to generate active force, a process that is fuelled by chemical energy from the hydrolysis of adenosine triphosphate (ATP). The active force acts to deform the muscle tissue, and so the energy of the sliding filaments is then stored as potential energy in the deformed tissues or spent doing external mechanical work. In the 3D muscle model, we simulated the increase in muscle energy via nervous system stimulation by prescribing a time-varying activation that increased the active energy. The active energy available to deform the tissue also depended on the local fibre stretches and velocities and was distributed and stored in the deformed tissue.

In the first paper in this series (Wakeling et al., 2020), we examined how energy is distributed across muscle tissue during fixed-end contractions where no external work was done when the muscle was fixed at its resting length. To do this, we used a quasistatic model formulation in which tissue kinetic energy was not accounted for, nor were the effects of strain rate on the active energy. Despite this, there were a number of similarities in the energy distribution across tissue components between the fixed-end quasistatic contractions in our previous study and the cyclic dynamic contractions reported here. As we activated the muscle, the active energy of the fibres became more negative as the fibres contracted (Figure 3A). This active energy was stored as potential energy in the deformed tissue, particularly the muscle base material that represents additional tissue within and surrounding the fibres, including the extracellular matrix. The volumetric energy, or the energy due to the muscle volume change, also increased as the active force became more negative as in Wakeling et al. (2020), but to a far lesser extent than the muscle base material energy.

In this study we additionally accounted for the kinetic energy of tissue mass and found that the kinetic energy-density (total energy across the tissue relative to the sum of the total muscle and aponeuroses volumes in J m^–3^) was greatest when the muscle was near its shortest and longest lengths (Figure 3B). Although the overall muscle velocity in the *x* direction was lowest at these times, the kinetic energy depends on the local tissue velocities that have components in all three directions, and so motion of the tissue in transverse directions (*y* and *z*) may account for the high kinetic energy when the muscle *x* velocity was low (Supplementary Figure 1). The local velocity also varies across the tissue: the tissue lags behind the motion of the non-fixed end due to its own inertia. The kinetic energy-density throughout a contraction cycle was greater for larger muscles with greater mass (Figure 4). In the quasistatic model that does not account for the effects of muscle mass and kinetic energy, the non-dimensional behaviour of the model, including the energy-density distribution, would be identical for models of different sizes. Accounting for kinetic energy creates a scale-dependent distortion such that models of different sizes but the same geometric proportions will have different distributions of energy-density. We found that the active energy-density was higher (less negative), the muscle base material energy-density was lower, and the aponeurosis energy-density was higher for larger compared to smaller muscles (Figure 4). Thus, larger muscles may make use of base material, or the extracellular matrix, less and aponeurosis more for energy storage and return during contraction than smaller muscles.

Both the quasistatic (Ryan et al., 2020; Wakeling et al., 2020) and dynamic model formulations do not account for energy dissipation in the form of heat. Muscles in living animals constantly dissipate heat, and the amount of thermal energy loss depends on a range of contractile factors, such as the activation, contraction speed and fibre-type properties of the muscle (Woledge et al., 1985). In our simulations, more energy may have been stored in the tissue or been available to do external work than if heat dissipation were accounted for in the model energy balance equation. However, it is unlikely that accounting for heat dissipation would have substantially altered our reported effects of muscle mass. Future work could aim to account for energy loss through heat dissipation in the formulation of 3D muscle models.

### Implications of Muscle Mass for Whole Muscle Function

Comparative studies have long sought to examine the maximum mechanical work or average mechanical power during cyclic contractions across animals of different sizes (Weis-Fogh and Alexander, 1977; Pennycuick and Rezende, 1984). The net mechanical work per cycle depends on the force a muscle generates over its change in length. Because muscle force scales with cross-sectional area, and muscle length change scales with optimal length, larger muscles will have higher absolute mechanical work per cycle than smaller muscles due to their larger cross-sectional areas and longer optimal lengths. However, since muscle force scales with cross-sectional area that has dimensions of length-squared, and the length change scales with optimal length which has dimensions of length, the work scales with the volume which has dimensions of length-cubed. Muscle tissue density is often considered to be constant across muscles, so the work per cycle also scales with muscle mass. Thus, based on these assumptions, two muscles with the same geometric proportions of different sizes or length scales would, in theory, generate the same mass-specific mechanical work per cycle. However, as muscle force and work are difficult to measure, particularly in larger animals, this theory has not been directly tested.

These earlier comparative studies, as with most studies in biomechanics, ignored the effects of muscle mass on contractile behaviour. While a muscle’s force increases in proportion to its area, or the length-squared, the muscle’s mass and acceleration increase in proportion to the volume, or length-cubed, and length, respectively. Thus, the loads due to muscle mass increase faster than the muscle force available to accelerate the tissue loads as muscles increase in size. As a consequence, the larger of the two muscles with the same geometric proportions but different sizes or length scales will have lower mass-specific mechanical work per cycle due to its greater muscle mass (Ross et al., 2018a, 2020). Submaximal fibre activation can amplify the effects of muscle mass, as evidenced by studies that have shown lower maximum shortening speeds during submaximal compared to maximal contractions of rat-sized muscle (Holt et al., 2014; Ross and Wakeling, 2016). In this study, we found that the reduction in mass-specific work due to muscle mass with greater muscle size is amplified with submaximal activation (Figure 5B), which is consistent with the results of previous 1D model simulations (Ross et al., 2018a). Therefore, muscle mass is an important determinant of whole muscle behaviour, particularly for larger muscles and during submaximal contractions.

Larger muscles may mitigate the effects of their greater mass by having different geometric proportions and architecture than smaller muscles. In this study, we found that greater initial pennation angle resulted in smaller reductions in mass-specific work per cycle with greater muscle size (Figure 5B). While data on scaling of muscle pennation angle with body sizes are limited, positive allometry for pennation angle has been shown for monitor lizard muscles across a range of body sizes (8 g short-tailed monitors to 40 kg Komodo dragons), in that larger animals have higher muscle pennation angles than smaller animals (Dick and Clemente, 2016). While we varied fibre length with the initial pennation angle of the muscle model, we geometrically scaled the model to larger sizes such that the initial fibre lengths were the same relative length across models of different sizes. However, muscles in larger animals tend to have relatively shorter fibres compared to muscles in smaller animals (Alexander et al., 1981; Pollock and Shadwick, 1994; Eng et al., 2008). While we found greater maximum fibre strains with greater initial pennation angles, this did not vary with scale, unlike pennation angle over time (Figure 6), so scaling fibre length with muscle size likely would not have altered our results. Thus, scaling of fibre length with body mass in living animals may be due to space constraints within a limb compartment rather than as a means to minimise the contractile consequences of muscle mass.

We found that the mean fibre pennation angle increased when both the muscle and fibres were shortening (Figure 6), which is consistent with previous *in vivo* ultrasound measures of human muscles during cyclic contractions (Kurokawa et al., 2001; Lichtwark et al., 2007; Wakeling et al., 2011; Dick and Wakeling, 2017; Randhawa and Wakeling, 2018). Rotating to higher angles during shortening allows the fibres to shorten slower than the muscle belly, and as a consequence, generate greater forces. However, this increase in force with higher fibre rotation is balanced by a force reduction due to the fibres no longer being oriented along the longitudinal axis of the muscle. We found smaller fibre rotations over a contraction cycle for larger muscles; however, this difference in fibre rotation with greater muscle size was smaller with higher initial pennation angle (Figure 6). Higher fibre angles during shortening may allow larger muscles to overcome the effects of their mass and achieve higher mass-specific work per cycle, although whether this relationship is causal cannot be determined from our simulations.

We may have seen different fibre strains and rotations had we constrained movement of the model aponeuroses. The bulging of muscle and movement of aponeuroses in transverse directions *in vivo* is limited by resistive forces applied by neighbouring muscles, connective tissue, and skin, which can alter muscle fibre strains and rotations (Wakeling et al., 2013; Ryan et al., 2019). In our simulations, we did not apply transverse compressive tractions to the muscle to mimic the effects of loads from surrounding tissue, as these loads acting on muscle during dynamic contractions are poorly understood. As a result, our muscle model and aponeuroses were free to bulge and rotate in ways that may not entirely reflect the behaviour of muscle *in vivo*. While the lack of transverse loads in our simulations may have altered the fibre strains and rotations, it is unlikely that they would have substantially influenced our reported effects of muscle mass, given that we previously found a similar pattern of mass effects across scales for a mass-enhanced 1D model.

Studies have shown that the properties of aponeurosis can alter fibre strains and rotations (Rahemi et al., 2015; Eng and Roberts, 2018), and so the properties of our aponeuroses may have also influenced the fibre strains across the different initial pennation angles in our simulations. To model the aponeuroses, we used two sheets of tissue that were uniform in thickness and composed of a base material with embedded collagen-like fibres. These fibres were unidirectional and oriented along the length of each aponeurosis at rest so that the overall behaviour of the tissue was anisotropic, consistent with previous experimental studies (Azizi and Roberts, 2009; Azizi and Roberts, 2009). We selected the thickness and material properties of the aponeuroses so that the longitudinal and transverse aponeurosis strains during maximal fixed-end contractions matched *in situ* measures of intact aponeurosis during the same contractile conditions (Azizi and Roberts, 2009). However, the utility of these *in situ* aponeurosis measures is limited, as only muscle forces acting externally and not forces applied to the aponeurosis can be measured or controlled. Thus, it is not clear to what extent our modelled aponeuroses reproduced the behaviour of *in vivo* aponeuroses, and so further work is needed to quantify the structural and material properties of aponeurosis and determine its role in constraining fibre strains and rotations during contraction.

The properties of aponeurosis may also vary with muscle scale, and this may have influenced our reported effects of muscle mass. Aponeurosis tissue, as well as tendon, likely plays an important role in energy storage and return during locomotion (Wager and Challis, 2016; Arellano et al., 2019). In our simulations, we assumed that the aponeurosis had the same relative effect on the model across different length scales or sizes, in that the stress-strain properties were constant, and the thickness, length, and width of the aponeuroses scaled with the length scale. While there is some evidence that tendon cross-sectional area relative to that of muscle varies with animal body mass (Alexander et al., 1981; Pollock and Shadwick, 1994), it is not yet known if the material or structural properties of aponeurosis vary with body and muscle size. It may be that the energetic role of these elastic structures varies with body size and alters the contractile effects of muscle mass.

### Effects of Tissue Properties on Cyclic Muscle Contractions

Whole muscles are typically considered to behave as single units where the strains and strain rates are uniform along the muscle’s length, and this assumption is reflected in 1D Hill-type models in which a single contractile element with a single length at any given time is used to represent the active muscle force (Zajac, 1989). In reality, whole muscles are composed of thousands of sarcomeres grouped into myofibrils at the microscopic level, which constitute muscle fibres at the cellular level that are then bundled into fascicles at the tissue level. Experiments on *in situ* and *in vivo* muscle during dynamic contractions have shown that fascicles within different regions of whole muscle can have different strains and be at different positions on their force-length curves at a given time (Pappas et al., 2002; Ahn et al., 2003, 2018; Soman et al., 2005; Shin et al., 2009).

In this study we found that tissue accelerations in the longitudinal direction varied along the muscle’s length for both the *in situ* and simulated contractions, and this non-uniformity in accelerations was more pronounced with greater muscle size or greater added mass (Figure 9). While the non-uniformity in muscle behaviour at the tissue level could be due to regional variations in activation (Monti et al., 2003; Rahemi et al., 2014) and myofascial force transmission (Tijs et al., 2015), in this study we show that the presence of muscle mass may also contribute to this non-uniformity during cyclic contractions.

Muscle mass may also have implications for non-uniformity at the microscopic level. Consider a massless ideal spring with uniform structural and material properties along its length. If one end of the spring is fixed and a tensile load is applied to the other, the spring’s response will be uniform in that the strain will be constant everywhere along its length. If the distributed mass of the spring is then accounted for, the spring’s response to the same tensile load will be different and will depend on the size and mass of the spring. Initially, the strains will be high near the applied load and low or zero near the fixed end. Then, a wave of strains will propagate along the length of the spring, causing a time delay between when the material near the free and fixed ends experience extension. Once the system settles, the strain across the spring’s length will become more uniform. This pattern of behaviour in a spring with mass, which has also been shown in simulations of a mass-enhanced 1D Hill-type model (Ross and Wakeling, 2016) and the 3D model in this study, is consistent with experimental findings of sarcomere strains across different regions of intact whole muscle. Moo et al. (2016) found that sarcomere strains are not uniform across different regions of intact muscle, and that the greatest sarcomere strains occur close to the myotendinous junction where external loads are applied. These strains become less uniform when muscle is activated during fixed-end contractions (Moo et al., 2017), but this non-uniformity in sarcomere strains decreases over time (Moo and Herzog, 2020). This pattern of sarcomere non-uniformity may be in part due to muscle mass and may vary depending on the size of the muscle, much like the response of the spring with mass and 1D and 3D muscle models that account for muscle mass.

### Conclusion

Over the last decade there has been growing interest in using 3D muscle models to explain the mechanisms that underly whole muscle contraction. As these models become more refined, they are increasingly able to replicate features of *in situ* and *in vivo* muscle behaviour. Rahemi et al. (2014) showed that muscle tissue activation results in S-shaped fibres during simulated fixed-end contractions, which is consistent with findings from *in vivo* measures of human medial gastrocnemius muscle (Namburete et al., 2011). More recently, we showed similar magnitudes and patterns of muscle bulging during submaximal contractions for the 3D model (Wakeling et al., 2020) as for *in vivo* muscle visualized using ultrasound (Randhawa et al., 2013) and magnetic resonance with diffusion tensor imaging (Wakeling et al., 2020). We also showed a similar reduction in muscle thickness and pennation angle with transverse compression (Ryan et al., 2020) as measures of *in vivo* human calf muscle (Ryan et al., 2019). In this study, in which we used a model formulation that accounts for distributed muscle mass and fibre force-velocity effects, we were again able to show similarities in behaviour between the simulated 3D model and experimental muscle. We found that greater muscle size, and therefore mass, for the simulations and greater added mass for the *in situ* experiments, as well as higher maximum strain amplitude, led to lower maximum and higher minimum acceleration in the longitudinal direction near the middle of the muscle compared to at the non-fixed end. Greater muscle size and higher maximum strain also led to greater reductions in mass-specific work per cycle, consistent with previous results from experiments on *in situ* rat plantaris muscle (Ross et al., 2020). This reduction in mass-specific work with larger muscle size was lower for simulated muscles with higher initial pennation angles. We also found that larger muscle size resulted in higher relative kinetic energy per cycle, relatively more energy stored in the aponeurosis, and less stored in the base material that represented the intra and extracellular tissue components apart from the myofibrils. While these results highlight that muscle mass can substantially decrease muscle performance, higher initial pennation angle and greater energy storage in elastic tissues may mitigate some of these performance losses.

## Nomenclature

**Activation** specifically refers to the active *state* of the contractile elements (muscle fibres) and is used to scale the active force that they can develop. In muscle physiology, **excitation** refers to the electrical potentials on the membrane of the muscle fibre that are typically recorded using EMG.

**Muscle contraction** is the *process* of muscle developing forces when its activation level is greater than zero. In muscle physiology, contraction does not necessarily mean shortening because tension can be developed without a change in length.

The **longitudinal direction** is the major *x*-axis of the muscle. This can be considered the direction that would be between the proximal and distal tendons in a fusiform muscle, and so it is in the commonly phrased “line-of-action.” We do not use “line-of-action” because forces and deformations occur in three dimensions in this study and so there is no unique line-of-action.

**Transverse direction** is used to describe directions in the *y–z* plane, and thus is perpendicular to the longitudinal direction of the muscle. This is sometimes called the radial direction in other studies.

**Muscle mass** refers to the total mass in kilograms of the muscle belly, including the muscle fibres, aponeuroses, and connective tissue. Note that this muscle mass is distributed throughout the tissue.

**Added mass** refers to the effective mass that we added to the *in situ* muscle at a single location along the muscle’s length. See the sections “Experimental Data Collection” and “Experimental Data Analysis” for further details. **Scale** refers to the length scale factor that multiplied the lengths of the muscle geometries to geometrically scale the muscle model to larger sizes. See the section “Muscle Model Geometries” for details of this scaling. Both increasing the added mass and scale increase the total mass of the muscle, although the effective mass in the *in situ* experiments was added only to a single location whereas increasing the model scale increased the mass distributed throughout the muscle and aponeurosis tissue.

## Data Availability Statement

The original contributions presented in the study are included in the article/Supplementary Material, further inquiries can be directed to the corresponding author.

## Ethics Statement

The animal study was reviewed and approved by the Faculty of Arts and Sciences Institutional Animal Care and Use Committee of Harvard University and the University Animal Care Committee of Simon Fraser University.

## Author Contributions

SR and JW contributed to the study design. SR, SD, NN, and JW contributed to the model development and reviewed the first draft of the manuscript. SR ran the model simulations, collected the experimental data, analysed the results, and wrote and revised the first draft of the manuscript. All authors contributed to the article and approved of the submitted version.

## Conflict of Interest

The authors declare that the research was conducted in the absence of any commercial or financial relationships that could be construed as a potential conflict of interest.

## Supplementary Material

Supplementary Figure 1 Local velocity of quadrature points over time. *x* **(A)**, *y* **(B)**, and *z* **(C)** components of the local velocity in the current direction (shading) of quadrature points as a function of their current *z* and *x* positions for a sample simulation with scale 1, initial pennation angle of 15.3°, maximum normalised excitation of 1, and maximum strain amplitude of 5%. We measured the local velocity in the current configuration at points that were in the *xz* plane at *y* = 0 in the initial configuration at times *t* = 0.125 s when the muscle was at its longest length, *t* = 0.25 s halfway through the shortening phase, *t* = 0.375 s when the muscle was at its shortest length, and *t* = 0.5 s when the muscle was at its initial length.

## Appendix

### Appendix 1: Details of Model Formulation

The model we used in this paper is based on the three-dimensional model introduced in Wakeling et al. (2020) to approximate the deformation of muscles during fixed-end contractions. We added inertial and velocity effects to this model to capture the dynamic behaviour of muscle tissue. The combined momentum and mass balance in the current configuration *V* of the muscle is as follows:

$$\frac{\partial \left(\rho \,\text{v}\right)}{\partial t}+\text{div}\,\left(\rho \,\text{v}⊗\text{v}\right)=\mathrm{div}\,\sigma \,\left(u,v,p,J\right),J-I_{3}\,\left(u\right)=0,p-\frac{\kappa }{2}\,\left(J-\frac{1}{J}\right)=0,$$

where ρ is the density of the muscle, **v** is the tissue velocity, **u** is the tissue displacement, σ is the Cauchy tensor, *J* is the dilation, *I*_3_ is the third invariant, *p* is the pressure, and κ is the bulk modulus. Bold symbols indicate vector variables and ⊗ denotes the usual outer product between two vectors. The kinetic energy *K*_int_ of the muscle in *V* is defined as:

$$K_{\text{int}}\,\left(v\right):=\frac{1}{2}\,\int_{V}\rho \,\text{v}\cdot \text{v}\,d\,V,$$

where “⋅” denotes the three-dimensional dot product. The muscle fibre stress relative to the maximum isometric stress ${\hat{\sigma }}_{\mathrm{mus},\mathrm{fibre}}$ depends on the isovolumetric stretch λ_iso_. Using the active stress-stretch ${\hat{\sigma }}_{\text{stretch}}\,\left(\lambda_{\text{iso}}\right)$, passive stress-stretch ${\hat{\sigma }}_{\text{pas}}\,\left(\lambda_{\text{iso}}\right)$ and stress-strain rate relationships ${\hat{\sigma }}_{\text{vel}}\,\left({\dot{\lambda }}_{\text{iso}}\right)$, ${\hat{\sigma }}_{\mathrm{mus},\mathrm{fibre}}$ is defined as:

$${\hat{\sigma }}_{\mathrm{mus},\mathrm{fibre}}\,\left(u,v\right):=\text{a}\,\left({\text{q}}_{0},t\right)\,{\hat{\sigma }}_{\text{stretch}}\,\left(\lambda_{\text{iso}}\right)\,{\hat{\sigma }}_{\text{vel}}\,\left({\dot{\lambda }}_{\text{iso}}\right)+{\hat{\sigma }}_{\text{pas}}\,\left(\lambda_{\text{iso}}\right)$$

where q_0_ is a point in the initial configuration of the muscle *V*_0_. The fibre stretch λ_iso_ is the length of the current orientation vector **a** and we define the strain rate in the current direction of the fibre ${\dot{\lambda }}_{\text{iso}}$ as:

$${\dot{\lambda }}_{\text{iso}}=\frac{a^{\text{T}}\text{dev}\left[\varepsilon \left(v\right)\right]a}{\lambda_{\text{iso}}},$$

where,

$$\text{dev}\left[\varepsilon \left(v\right)\right]=\varepsilon \left(v\right)-\frac{1}{3}\text{div}\left(v\right)I,\varepsilon \left(v\right)=\frac{1}{2}\left(\nabla v+\nabla v^{T}\right).$$

The tensor ε(**v**) is the strain rate tensor and **I** is the identity matrix. The active stress tensor is defined as in Wakeling et al. (2020) but with the added term from ${\hat{\sigma }}_{\text{vel}}$ such that:

$${\hat{\sigma }}_{\text{act}}\left(u,v\right):={\hat{\sigma }}_{\mathrm{mus},\mathrm{fibre}}a⊗a.$$

The numerical implementation of the momentum and mass balance is semi-implicit in time, in which the velocity **v** is updated explicitly, whereas the displacement **u** is implemented implicitly. As such, the implementation ${\hat{\sigma }}_{\text{act}}$ only depends on the current state of **u** (computed via the finite element method) and **u** at the previous time step. We implemented the model in the finite element library deal.II version 8.5.1 (Arndt et al., 2017) using the tutorial step-44 (Pelteret and McBride, 2012; Pelteret, 2014).

### Appendix 2: Details of Model Geometries

**TABLE 1:** Initial dimensions of model geometries.

**Scale**	**α_0_ (°)**	**β_0_ (°)**	**Muscle length (m)**	**Muscle width (m)**	**Fibre length (m)**	**Aponeurosis length (m)**	**Aponeurosis thickness (m)**
1	15	11.5	0.27	0.055	0.06366284	0.208	0.003
2.5	15	11.5	0.675	0.1375	0.15915709	0.52	0.0075
3.5	15	11.5	0.945	0.1925	0.22281993	0.728	0.0105
1	20	15.3	0.27	0.055	0.065	0.208	0.003
2.5	20	15.3	0.675	0.1375	0.1625	0.52	0.0075
3.5	20	15.3	0.945	0.1925	0.2275	0.728	0.0105
1	25	19.0	0.27	0.055	0.06677424	0.208	0.003
2.5	25	19.0	0.675	0.1375	0.1669356	0.52	0.0075
3.5	25	19.0	0.945	0.1925	0.23370983	0.728	0.0105
1	30	22.7	0.27	0.055	0.06902991	0.208	0.003
2.5	30	22.7	0.675	0.1375	0.17257477	0.52	0.0075
3.5	30	22.7	0.945	0.1925	0.24160468	0.728	0.0105
