# Supplementary figures and images for: The Energy of Muscle Contraction. III. Kinetic Energy During Cyclic Contractions
Source: Front Physiol. 2021 Apr 7;12:628819. doi: 10.3389/fphys.2021.628819 (PMC8058367; doi:10.3389/fphys.2021.628819)

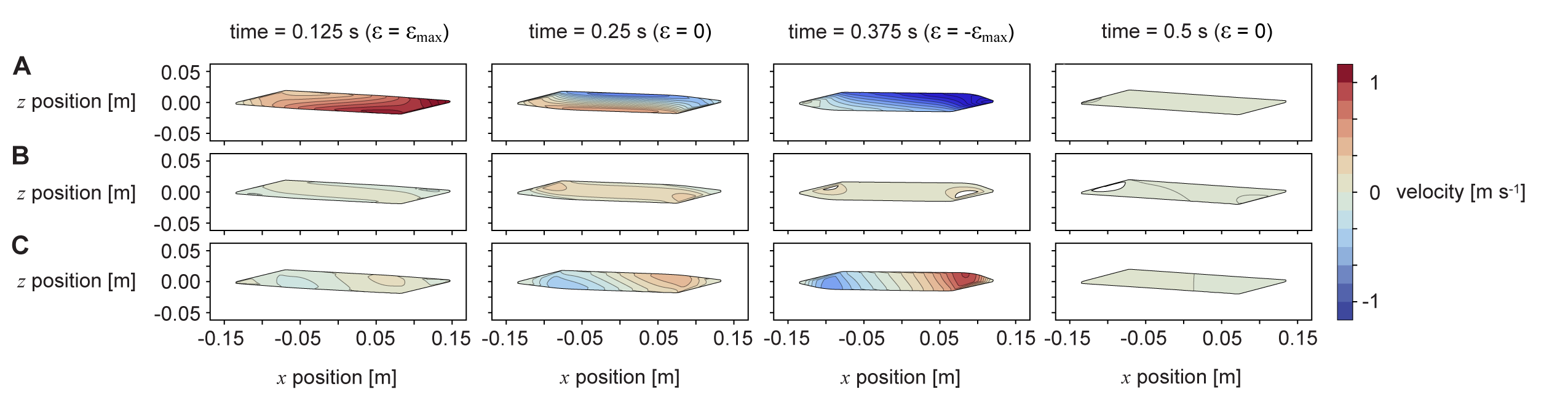

Supplement: Supplementary Figure 1 — Local velocity of quadrature points over time. x (A), y (B), and z (C) components of the local velocity in the current direction (shading) of quadrature points as a function of their current z and x positions for a sample simulation with scale 1, initial pennation angle of 15.3°, maximum normalised excitation of 1, and maximum strain amplitude of 5%. We measured the local velocity in the current configuration at points that were in the xz plane at y = 0 in the initial configuration at times t = 0.125 s when the muscle was at its longest length, t = 0.25 s halfway through the shortening phase, t = 0.375 s when the muscle was at its shortest length, and t = 0.5 s when the muscle was at its initial length. [file Image_1.TIF]
